# Vitamins in the Prevention and Support Therapy of Neurodegenerative Diseases

**DOI:** 10.3390/ijms26031333

**Published:** 2025-02-04

**Authors:** Karolina Orywal, Katarzyna Socha, Piotr Iwaniuk, Piotr Kaczyński, Jakub Ali Farhan, Wojciech Zoń, Bożena Łozowicka, Maciej Perkowski, Barbara Mroczko

**Affiliations:** 1 Department of Biochemical Diagnostics, Medical University of Bialystok, Waszyngtona 15A, 15-269 Bialystok, Poland; mroczko@umb.edu.pl; 2 Department of Bromatology, Medical University of Bialystok, Mickiewicza 2D, 15-222 Bialystok, Poland; katarzyna.socha@umb.edu.pl; 3 Institute of Plant Protection—National Research Institute, Chełmońskiego 22, 15-195 Bialystok, Poland; p.iwaniuk@iorpib.poznan.pl (P.I.); p.kaczynski@iorpib.poznan.pl (P.K.); bozena.lozowicka@gmail.com (B.Ł.); 4 Department of Public International Law and European Law, University of Białystok, Mickiewicza 1, 15-213 Białystok, Poland; j.farhan@uwb.edu.pl (J.A.F.); w.zon@uwb.edu.pl (W.Z.); m.perkowski@uwb.edu.pl (M.P.); 5 Department of Neurodegeneration Diagnostics, Medical University of Bialystok, Waszyngtona 15A, 15-269 Bialystok, Poland

**Keywords:** neurodegenerative diseases, brain function, cognitive function, neuroprotection, dementia prevention, nutrients, diet, food law, EU legislation

## Abstract

Neurodegenerative diseases such as Alzheimer’s disease (AD), Parkinson’s disease (PD), amyotrophic lateral sclerosis (ALS), and multiple sclerosis (MS), which are a consequence of the progressive loss of neuronal function and structure, cause significant cognitive impairment. The incidence of these diseases in the world’s population is constantly increasing as a result of an aging population. Although genetic and environmental factors are most often mentioned as the pathogenetic factors of these diseases, increasing evidence points to the important role of proper nutrition in the prevention and support of the treatment of these disorders. A healthy, balanced diet can mitigate the risks associated with the risk factors mentioned above and slow the progression of the disease by reducing oxidative stress and inflammation. Vitamins B, D, E, C, K, and A have been shown to support cognitive functions and protect the nervous system. This review demonstrates the importance of vitamins in preventing and supporting the therapy of neurodegenerative diseases. Information regarding the health-promoting properties of these vitamins must be effectively communicated to consumers seeking to protect their health, particularly in the context of neurodegenerative diseases. Consequently, this review also examines the authorized health claims under EU food law related to these vitamins, assessing their role in promoting awareness of the vitamins’ potential benefits for neuroprotection and the management of neurodegenerative diseases.

## 1. Introduction

In recent years the incidence of neurodegenerative diseases such as Alzheimer’s disease (AD), Parkinson’s disease (PD), amyotrophic lateral sclerosis (ALS), and multiple sclerosis (MS)—all characterized by the progressive gradual degeneration and death of neurons —continues to rise. Currently, around 25 million people globally are affected by dementia associated with a variety of neurodegenerative diseases. As a result of the ever-increasing life expectancy, it is expected that by 2050 the number of people suffering from these diseases will exceed 115.4 million [1]. In response to these challenges, national governments and international organizations are undertaking various initiatives to expand knowledge and to prevent or delay the progression of neurodegenerative diseases. A prominent example of such efforts is the EU Joint Programme—Neurodegenerative Disease Research (JPND), a collaborative research program aimed at coordinating the research efforts of EU member states and associated countries in combating neurodegenerative diseases [2].

The risk of neurodegenerative diseases is often associated with genetic, environmental, and lifestyle factors, in particular physical inactivity, smoking, alcohol consumption, and poor nutrition [3]. These diseases are associated with the occurrence of devastating cognitive and motor function disorders, which significantly impair the quality of life of patients [4]. Neurodegenerative diseases are also often associated with the occurrence of malnutrition and dysphagia, both of which significantly impact an individual’s nutritional health. Oropharyngeal dysphagia makes it difficult for patients with neurodegenerative diseases to consume food and maintain proper hydration, which is the most important cause of malnutrition in this group of patients. In turn, weight loss and malnutrition may intensify neurodegenerative processes, accelerating the progression of the disease. As a consequence, the patient’s quality of life deteriorates and mortality increases [5].

**Figure 1:**
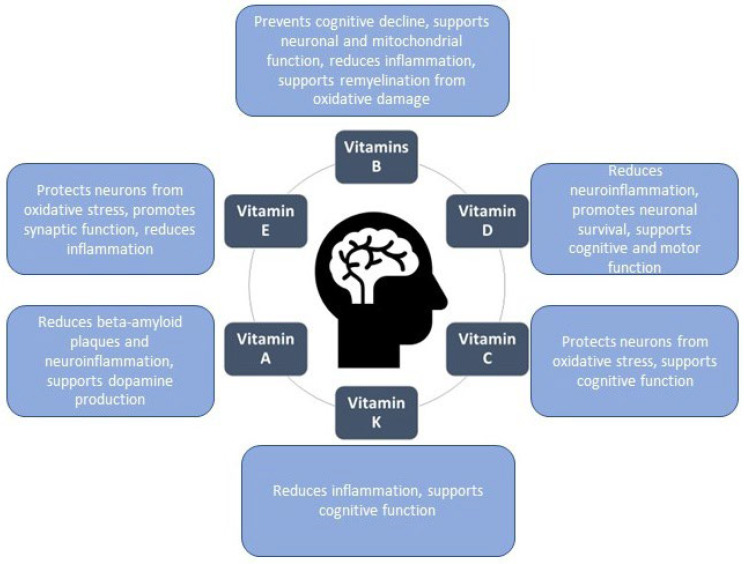
Vitamins with beneficial effects on the human brain.

Numerous studies, widely discussed in the literature, demonstrate that diet plays a critical role in both prevention and supportive therapy. Proper nutrition can offer protective effects and may even slow disease progression by mechanisms such as reducing oxidative stress, minimizing inflammation, and supporting neuroplasticity. The human diet contains nutrients in various proportions, including carbohydrates, proteins, fats, vitamins, and minerals. Vitamins play a significant role in supporting brain and nervous system health, and some of them have been implicated in preventing, treating, and potentially slowing neurodegenerative diseases (Figure 1) [6,7,8]. These pieces of information are of considerable importance to consumers, as they enable them to make more informed purchasing decisions that take into account the impact of diet on the risk of neurodegenerative diseases. By understanding the protective effects of proper nutrition—such as its role in reducing oxidative stress, minimizing inflammation, and supporting neuroplasticity—individuals can make dietary choices that align with their health goals. Moreover, awareness of the specific roles that vitamins and other nutrients play in brain health empowers consumers to select products that may contribute to the prevention, management, or slowing of neurodegenerative disease progression, thereby fostering a more proactive approach to their health. Therefore, it is crucial that, in addition to education, legal instruments are used to communicate such information effectively [9]. In the context of food labeling, this primarily involves the use of health claims. By using legally authorized health claims, consumers are provided with scientifically substantiated information, enabling them to make informed decisions about their diet and its potential impact on long-term brain health.

This review aims to present the role of vitamins such as B vitamins and vitamins D, E, A, C, and K in the prevention, treatment, and potential support of the therapy of neurodegenerative diseases. The analysis of the importance of vitamins in maintaining neuronal health, inhibiting oxidative stress, and regulating neurotransmitters presented in this paper shows how vitamin deficiencies or supplementation may influence the progression of neurodegenerative diseases and whether vitamins provide neuroprotective benefits, slowing the decline of cognitive and motor functions. Additionally, this review also examines the authorized health claims under EU food law related to these vitamins, assessing their role in promoting awareness of the potential benefits these vitamins offer for neuroprotection and the management of neurodegenerative diseases. By analyzing the legal framework surrounding these claims, this review highlights how such claims can contribute to consumer knowledge and guide informed decisions about the role of diet in preventing or managing neurodegenerative diseases.

## 2. Methods

**Figure 2:**
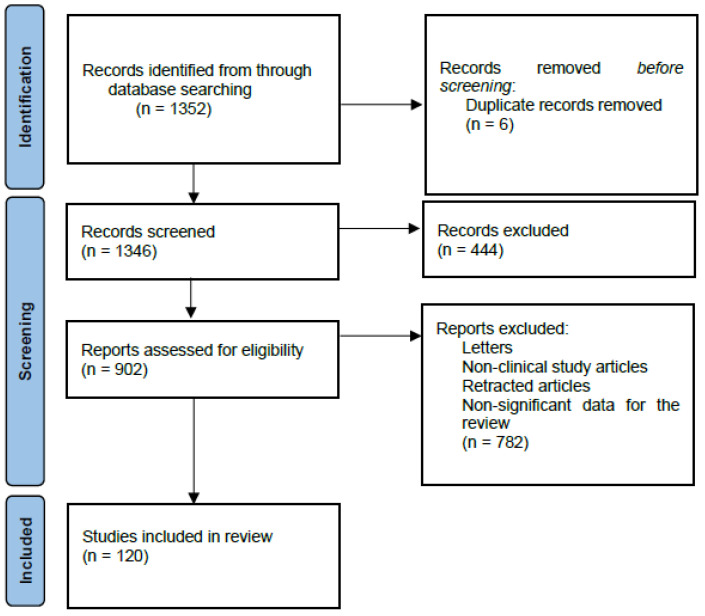
PRISMA 2020 flow diagram depicting methods for including studies in the review.

We performed a comprehensive literature search in the MEDLAB/PubMed electronic database for the last 5 years using the keywords “vitamins and neurodegenerative diseases” (n = 1352). The next step involved choosing vitamins essential for the review. Additionally, we used keywords related to specific neurodegenerative diseases: “Alzheimer’s Disease and vitamins” (n = 977), “Parkinson’s Disease and vitamins” (n = 539), “Amyotrophic Lateral Sclerosis and vitamins” (n = 104), “Multiple Sclerosis and vitamins” (n = 688), “Vitamins B and neurodegenerative diseases” (n = 236), “Vitamin C and neurodegenerative diseases” (n = 87), “Vitamin D and neurodegenerative diseases” (n = 228), “Vitamin E and neurodegenerative diseases” (n = 90), “Vitamin A and neurodegenerative diseases” (n = 72), and “Vitamin K and neurodegenerative diseases” (n = 20). Only full-text publications in English limited to studies on humans were selected. In the next step, letters, non-clinical study articles, retracted articles, and non-significant data for the review were excluded (Figure 2).

## 3. Vitamins in Neurodegenerative Diseases

### 3.1. Water-Soluble Vitamins

#### 3.1.1. Vitamins B

B vitamins, mainly B1 (thiamine), B2 (riboflavin), B3 (niacin), B6 (pyridoxine), B7 (biotin), B9 (folate), and B12 (cobalamin), play essential roles in maintaining brain health and preventing neurodegenerative diseases [8,10]. They support key metabolic processes such as DNA synthesis, nerve function, and the regulation of homocysteine levels as a risk factor for neurodegenerative disorders.

Vitamin B1 is a water-soluble vitamin that is necessary for proper energy metabolism and the functioning of the nervous system. In turn, its role in the conduction of nerve impulses, phosphorylation, and activation of chloride channels in neuronal membranes is crucial in the processes of energy production in the brain and maintaining neuronal health. Moreover, with the participation of thiamine, acetylcholine is synthesized, a neurotransmitter necessary in the processes of remembering and learning [11]. It has been shown that vitamin B1 deficiency leads to serious neurological disorders and contributes to the progression of neurodegenerative diseases due to the disruption of energy production in neurons, which in turn induces oxidative stress and leads to peripheral nerve inflammation and encephalopathy. In addition, reduced levels of vitamin B1 have been associated with memory disorders and the formation of amyloid plaques, which disturb the flow of calcium ions, ultimately leading to the death of neurons in the hippocampus and cerebral cortex, and thus contributing to the development of neurodegenerative diseases. Vitamin B1 deficiency also causes deterioration of cognitive functions, and some studies suggest that supplementation with vitamin B1 or its derivatives may improve memory and cognitive performance in patients with AD [12]. In addition, vitamin B1 also reduces oxidative stress, the main factor causing the degeneration of dopamine-producing neurons in Parkinson’s disease. It has been shown that adequate levels of vitamin B1 can help protect neurons and slow the progression of the disease. In turn, early research indicates that high doses of vitamin B1 may help alleviate motor symptoms in some patients with Parkinson’s disease [13]. In ALS, important etiological factors are mitochondrial dysfunction and oxidative stress; vitamin B1 deficiency may exacerbate these processes. Vitamin B1 supports the function of mitochondria, which is crucial for energy production in neurons, potentially having neuroprotective effects [14]. No adverse effects have been reported from consuming vitamin B1 with food or dietary supplements. When the intake of this vitamin exceeds 5 mg, its absorption is reduced and the excess is excreted in the urine [15].

Riboflavin (vitamin B2) is a precursor in the synthesis of flavin adenine dinucleotide (FAD) and flavin mononucleotide (FMN), which in turn are essential cofactors in the electron transport chain, proper functioning of mitochondria, and antioxidant defense [16]. It also helps regenerate glutathione, which acts as an antioxidant and protects neurons against oxidative damage. Additionally, glutathione plays an important role in the synthesis and maintenance of myelin, which protects the surrounding nerve fibers. Vitamin B2 deficiencies may induce oxidative stress, which negatively affects the functioning of the nervous system, and may result in neurological symptoms such as peripheral neuropathy, muscle weakness, and cognitive disorders. Due to its involvement in the proper functioning of mitochondria and its antioxidant properties, vitamin B2 is considered a potential therapeutic agent in the treatment of neurodegenerative diseases such as Parkinson’s disease, multiple sclerosis, ALS, and Alzheimer’s disease [17]. Some studies have shown that consuming more than 1.8 mg/day of this vitamin has a positive effect on cognitive functions and visual perception [18].

Vitamin B3 also plays an important role in the prevention of neurodegenerative diseases, being a precursor to the production of NAD+ (nicotinamide adenine dinucleotide) and NADP+ (nicotinamide adenine dinucleotide phosphate), which play a key role in energy metabolism in neurons, participate in DNA repair and antioxidant processes, and help protect neurons against oxidative stress, which is a common cause of cell death in these disorders [19]. Reducing NAD+ levels is associated with neuronal dysfunction that progresses with age. Vitamin B3 is involved in the proper functioning of the immune system, reducing neuroinflammation, which is characteristic of the progression of neurodegenerative diseases such as Alzheimer’s disease and multiple sclerosis [20,21]. Moreover, this vitamin inhibits the production of pro-inflammatory cytokines and supports the repair mechanisms taking place in brain tissues. Vitamin B3 deficiency has been associated with symptoms of dementia, such as memory impairment, confusion, and mood changes [22]. Vitamin B3 may have a protective effect by inhibiting the formation of amyloid-beta plaques, a characteristic pathological change of Alzheimer’s disease. Some studies suggest that higher dietary intake of vitamin B3 may reduce the risk of developing Alzheimer’s disease and improve cognitive functions [21]. Adequate levels of vitamin B3 have also been linked to increased levels of sirtuins (SIRT1), proteins that help protect neurons and regulate inflammation and metabolism, slowing down neurodegeneration in Parkinson’s disease [23]. Moreover, vitamin B3 may partially prevent damage in demyelinating diseases such as multiple sclerosis by reducing inflammation and participating in the repair of the myelin sheath [24]. Vitamin B3, especially in NAD+-enhancing forms, is a promising therapeutic strategy for various neurodegenerative diseases by supporting mitochondrial function, reducing inflammation, and protecting neurons from oxidative damage [25].

Vitamin B6 is crucial for brain health because it participates in the production of important neurotransmitters, regulates homocysteine metabolism, and is involved in the regulation of oxidative stress. Vitamin B6 acts as a coenzyme in the synthesis of neurotransmitters such as serotonin, dopamine, GABA, and norepinephrine, which are involved in the regulation of mood, cognitive, and motor functions [26]. This makes vitamin B6 essential for the overall functioning of the nervous system. Vitamin B6 deficiency causes deterioration of cognitive functions and memory problems, especially in older people. Additionally, vitamin B6 is involved in the conversion of homocysteine into cysteine, thereby reducing the accumulation of homocysteine. It is harmful to neurons and its high level is associated with an increased risk of neurodegeneration, including Alzheimer’s disease, progression of Parkinson’s disease, and cognitive disorders [27]. Modica et al. suggest that people with Parkinson’s disease are much more likely to have low levels of vitamin B6, which may cause polyneuropathy, epilepsy, and other neurological complications in these patients [28]. Some studies also indicate that vitamin B6 supplementation, especially in combination with other B vitamins, may reduce brain atrophy in the hippocampus that occurs in patients with Alzheimer’s disease [29]. On the other hand, it has been shown that after taking large daily doses of this vitamin (>500 mg/day) and/or longer treatment (>6 months), neurological side effects may occur. Such situations are rare and usually reversible. Exposure to toxic doses of vitamin B6 may cause primary damage to nerve cell bodies, necrosis, and degeneration of axons and myelin. If the damage is not too advanced, after withdrawing vitamin B6, the nerves can regenerate and the symptoms disappear completely [30].

Vitamin B7, also known as biotin, is crucial for metabolism as a coenzyme for carboxylases that aid in synthesizing fatty acids, amino acids, and glucose. While its role in neurodegenerative diseases is less studied compared to other B vitamins like B6, B9, and B12, emerging evidence suggests biotin may offer neuroprotective benefits, particularly in multiple sclerosis and other demyelinating disorders. The positive effects of high-dose vitamin B7 in progressive MS are thought to stem from the increased activity of biotin-dependent enzymes involved in myelin fatty acid synthesis and mitochondrial energy metabolism [31]. These processes help preserve neuronal function, reducing the risk of neurodegeneration and vitamin B7 may also provide anti-inflammatory benefits through cGMP modulation in the cerebral microvasculature, oligodendrocyte differentiation, and Schwann cell production of neurotrophic factors, which are believed to help manage MS [32]. Further, it contributes to epigenetic regulation by promoting histone biotinylation, which affects gene expression critical for neuronal health and neurodegeneration prevention [33]. Aissa et al. suggest that the antioxidant properties of vitamin B7 help reduce oxidative stress in neurons, a key factor in the progression of neurodegenerative diseases like Alzheimer’s disease, Parkinson’s disease, and ALS. Although the direct connection between vitamin B7 and Alzheimer’s disease is limited, its role in energy metabolism and neuronal function suggests potential protective effects. Neurons rely heavily on energy for synaptic activity, and impaired metabolism of these cells is a hallmark of Alzheimer’s disease. In neurons, cGMP supports survival and plasticity, with hippocampal long-term potentiation requiring nitric oxide synthesis, which activates CREB through the cGMP-PKG pathway. In Alzheimer’s disease, amyloid-beta disrupts this mechanism by inhibiting sGC activity, but agents that elevate cGMP levels have been shown to restore long-term potentiation in rodent models [34]. Given cGMP’s neuroprotective effects, high-dose vitamin B7 may hold promise in preventing and managing Alzheimer’s disease. Similarly, although evidence is limited, antioxidant capacity and support of mitochondrial function of vitamin B7 may help protect neurons from the oxidative stress and dysfunction seen in Parkinson’s disease [32].

Vitamin B9, i.e., folic acid, plays a key role in the development of the nervous system and proper functioning of the brain. It is necessary for DNA synthesis, cell repair, methylation, and homocysteine regulation. Vitamin B9 is crucial for maintaining synaptic plasticity, which supports learning and memory. Vitamin B9 deficiency causes fetal neural tube defects and impairs the functioning of the nervous system by reducing the synthesis of acetylcholine. In turn, its deficiency leads to neuronal degeneration, which may result in cognitive disorders [35]. Vitamin B9 deficiency is more common in older people and results in neuronal damage, memory impairment, cognitive decline, dementia, and mood disorders [36]. Research indicates that adequate levels of vitamin B9 may protect against neurodegenerative diseases such as AD, PD, and multiple sclerosis [37]. Vitamin B9, in addition to vitamins B6 and B12, is involved in the conversion of homocysteine into methionine, and an increased level of homocysteine is associated with a greater risk of neurodegenerative diseases and vascular dementia. Homocysteine has neurotoxic effects and is involved in oxidative stress, inflammation, and neuronal damage [27]. Additionally, vitamin B9 is crucial for DNA methylation, which regulates gene expression and protects neurons, helping to prevent cognitive decline. It is also involved in producing neurotransmitters like serotonin, dopamine, and norepinephrine, which are essential for mood regulation, cognition, and neural communication. Vitamin B9 reduces oxidative stress and supports mitochondrial health, both key factors in preventing neurodegeneration. Vitamin B9 deficiency can increase amyloid-beta plaque buildup, a key marker in Alzheimer’s disease [38]. Low vitamin B9 levels have been linked to a higher risk of Parkinson’s disease, as elevated homocysteine can accelerate dopaminergic neuron loss, worsening motor symptoms. The role of vitamin B9 in dopamine synthesis is particularly relevant in Parkinson’s, where dopamine levels are diminished [39]. Its neuroprotective properties, such as reducing oxidative stress and aiding DNA repair, may offer therapeutic benefits in slowing degeneration in ALS, as well as supporting mitochondrial function, which is compromised in the disease, potentially slowing its progression [40]. In MS, vitamin B9 may provide anti-inflammatory and neuroprotective benefits and promote remyelination as MS involves immune-driven damage to the myelin sheath [41].

Vitamin B12 is involved in the development and maintenance of the nervous system, influencing memory, concentration, and cognitive functions [42]. It plays a key role in DNA synthesis, myelin formation, homocysteine metabolism, and neurotransmitter regulation. Deficiency of this vitamin can lead to myelopathy and neuropathy, which result from progressive demyelination of white matter in the brain and spinal cord. Insufficient vitamin B12 impairs myelin production, causing nerve damage and neurodegenerative symptoms such as cognitive decline and motor dysfunction [43]. Cecchetti et al. indicate that vitamin B12 can also help restore the integrity of the blood–brain barrier, the disruption of which is associated with neurodegenerative diseases [44]. In addition, vitamin B12 in combination with vitamins B6 and B9 helps reduce the level of homocysteine, the mentioned substance toxic to neurons. Homocysteine increases oxidative stress and inflammation and is therefore a greater risk of neurodegenerative diseases such as Alzheimer’s disease and vascular dementia [27]. In addition, vitamin B12 is necessary for the synthesis and repair of DNA, which ensures the proper development and functioning of neurons. Its deficiency may lead to DNA damage, which additionally contributes to neuronal degeneration and cognitive disorders. Moreover, vitamin B12 is involved in the synthesis of neurotransmitters such as serotonin, dopamine, and noradrenaline, which are necessary for the regulation of mood, cognitive functions, and motor control [10].

Vitamin B12 deficiency causes cognitive decline, memory impairment, and dementia, which are characteristic symptoms of Alzheimer’s disease. Some research suggests that vitamin B12 supplementation, along with folic acid and vitamin B6, may help lower homocysteine levels, potentially slowing cognitive decline and reducing brain atrophy in Alzheimer’s disease patients [45]. In Parkinson’s disease, vitamin B12 is important for protecting dopaminergic neurons that are gradually lost, and its supplementation may help reduce neuronal loss and improve motor function [46]. Bagur et al. demonstrated that vitamin B12 supplementation can help promote remyelination, reduce neuroinflammation, and alleviate symptoms such as fatigue and cognitive deficits in patients with multiple sclerosis [47]. In amyotrophic lateral sclerosis, there is degeneration of motor neurons and progressive muscle weakness. Some clinical trials have examined the use of high-dose methylcobalamin (a form of vitamin B12) as a potential therapy. A slight improvement in motor neuron survival and disease progression has been demonstrated [48].

#### 3.1.2. Vitamin C

Vitamin C, or ascorbic acid, is an essential antioxidant that plays a key role in brain health. In recent years, its involvement in neurodegenerative diseases has gained much attention. The involvement of this water-soluble vitamin in the process of neuroprotection, neurotransmitter synthesis and regulation of oxidative stress has been demonstrated. The factors mentioned are crucial in the prevention and treatment of neurodegenerative disorders [49,50]. Vitamin C is one of the most powerful antioxidants in the brain. Thanks to it, free radicals and reactive oxygen species (ROS) are neutralized, which protects neurons against oxidative damage. This protective effect may slow the progression of diseases such as AD and PD. Vitamin C is also involved in the production of neurotransmitters such as dopamine, noradrenaline, and serotonin, which are important in the regulation of mood, cognitive functions, and motor control [51]. Additionally, vitamin C plays a role in collagen synthesis, which is important for maintaining the structural integrity of blood vessels and connective tissue in the brain. In addition, it supports the integrity of the blood–brain barrier, which protects the brain against toxins and inflammation. Violation of this barrier is a common feature of many neurodegenerative diseases [52]. In addition, vitamin C maintains the health of neurons by participating in their repair, promoting the formation of the extracellular matrix. It also helps regulate the concentration of metal ions in the brain, especially iron. Dysregulation of iron levels is associated with oxidative stress and protein aggregation, which occur in the course of neurodegenerative diseases [53]. Vitamin C, by preventing excessive iron accumulation, reduces oxidative damage and provides neuroprotection [54]. It also helps fight inflammation of the nervous system, which contributes to the development of multiple sclerosis and Alzheimer’s disease. Some studies suggest that higher levels of vitamin C may improve memory and cognitive function in AD patients due to reduced oxidative stress and neuroinflammation [55]. Additionally, vitamin C participates in the removal of beta-amyloid plaques, thus reducing their toxic effect on neurons [56]. Research has shown that maintaining adequate vitamin C levels through diet or supplementation can help reduce the risk of neurodegenerative diseases and support overall brain health [57].

### 3.2. Fat-Soluble Vitamins

#### 3.2.1. Vitamin D

Vitamin D is crucial for maintaining brain health and has garnered attention for its potential neuroprotective effects in neurodegenerative diseases due to its roles beyond calcium regulation and bone health. It affects neuronal health, neuroinflammation, immune modulation, and oxidative stress, all of which are critical in developing neurodegenerative conditions [58]. Receptors for vitamin D are present on both neurons and glial cells in key areas of the brain, where this vitamin influences the differentiation and maturation of neurons and regulates the production of nerve growth factor (NGF) and glial cell line-derived neurotrophic factor (GDNF) [59]. These growth factors promote neuronal survival, growth, and repair. Additionally, vitamin D aids in the synthesis of important neurotransmitters, including acetylcholine, dopamine, and gamma-aminobutyric acid (GABA), which are essential for cognitive and motor functions [60]. Vitamin D helps regulate brain calcium levels, which are vital for proper neuronal signaling, as dysregulated calcium can lead to neuronal dysfunction and death. Its anti-inflammatory properties and ability to modulate immune responses make it protective in multiple sclerosis and Alzheimer’s disease, where inflammation is a key factor in disease progression [61]. As an antioxidant, vitamin D also helps counter oxidative stress, which contributes to neuronal damage and degeneration. Low vitamin D levels have been linked to cognitive decline and an increased risk of Alzheimer’s disease. Deficiency of this vitamin may contribute to the accumulation of beta-amyloid plaques, and worsen inflammation [62]. Furthermore, vitamin D supports synaptic plasticity and protects against neuroinflammation, which may help delay cognitive impairment in AD patients. In Parkinson’s disease, vitamin D plays a protective role for dopaminergic neurons, which are progressively lost. Pignolo et al. suggest that higher vitamin D levels are associated with a lower risk of developing PD, and supplementation of this vitamin may improve motor function and protect against dopaminergic degeneration [63]. Vitamin D’s ability to regulate immune responses and reduce neuroinflammation has also been linked to a reduced risk of developing MS and lower relapse rates in these patients. Adequate vitamin D levels may slow the progression of MS by reducing inflammation and supporting remyelination, further demonstrating its neuroprotective potential [64]. On the other hand, increased calcium levels caused by excess vitamin D can affect many organs, including the brain, causing vascular calcification. This may result in an increased risk of stroke and deterioration of cognitive functions. Therefore, regulating vitamin D intake is essential for maintaining vascular health and the functioning of the nervous system [65].

#### 3.2.2. Vitamin E

Vitamin E is a fat-soluble antioxidant that plays a key role in protecting the brain against oxidative stress. The importance of this vitamin has been studied for its potential benefits in the treatment of various neurodegenerative diseases. Its most active form, alpha-tocopherol, is essential for maintaining neuronal health by neutralizing free radicals, which are the main factors contributing to neurodegeneration [66]. Excessive free radicals cause damage to proteins, lipids, and DNA in brain cells, which leads to deterioration of cognitive functions and motor dysfunctions. Vitamin E supports the survival, growth, and repair of neurons by supporting neurotrophic factors, which alleviates neurodegenerative processes. Vitamin E deficiency increases oxidative stress, accelerating neuron loss processes, which is associated with the symptoms of neurodegenerative diseases. Low levels of vitamin E are associated with a greater risk of cognitive disorders, dementia, and Alzheimer’s disease, especially in older people [67]. Oxidative damage caused by free radicals accelerates the formation of beta-amyloid plaques and neurofibrillary tangles in Alzheimer’s disease [68]. In Parkinson’s disease, oxidative stress significantly contributes to the degeneration of dopaminergic neurons in the substantia nigra, which is responsible for motor control [69]. Vitamin E protects the lipid bilayers of neuronal membranes, preventing the oxidation of polyunsaturated fatty acids in them. This role is crucial for maintaining the integrity and function of nerve cells [66]. In addition to its antioxidant role, vitamin E has anti-inflammatory properties, which is particularly important in neuroinflammatory conditions such as Alzheimer’s disease and multiple sclerosis [70]. In diseases such as Alzheimer’s and Parkinson’s, vitamin E may help reduce the aggregation of proteins (e.g., beta-amyloid and alpha-synuclein), which protects neurons from the accumulation of toxins. Nolan et al. indicate that increased levels of vitamin E are associated with a slower rate of cognitive function deterioration in patients with Alzheimer’s disease [71]. Some clinical studies suggest that vitamin E supplementation may slow the progression of Alzheimer’s disease by reducing oxidative damage, reducing inflammation, and preventing the build-up of beta-amyloid plaques. This improves synaptic functions and neuroplasticity, which are necessary for maintaining memory and cognitive functions [71,72]. Although the evidence is mixed, some research suggests that a higher dietary intake of vitamin E may reduce the risk of developing Parkinson’s disease. In turn, its supplementation may potentially alleviate motor symptoms by reducing oxidative stress in dopaminergic neurons [69,73]. The results of research on the role of vitamin E in slowing down the progression of ALS are ambiguous, but they suggest a moderate neuroprotective effect related to its antioxidant properties [74]. Similarly, in multiple sclerosis, oxidative stress contributes to demyelination. The properties of vitamin E reduce oxidative damage, improving myelin function and providing therapeutic benefits to patients with multiple sclerosis [75]. Genetic variations have been found to influence the absorption and metabolism of vitamin E, potentially altering its overall impact. Recent research suggests that vitamin E supplementation may have detrimental effects on Alzheimer’s disease by promoting increased amyloid β synthesis. These diverse outcomes may explain the inconsistent findings related to its supplementation and emphasize the need for cautious use [76].

#### 3.2.3. Vitamin A

Vitamin A, a fat-soluble vitamin primarily recognized for its roles in vision and immune function, is also essential for brain health and has garnered attention for its potential in addressing neurodegenerative diseases. The active form of vitamin A, retinoic acid, is integral to neurodevelopment, synaptic plasticity, neurotransmission, and gene regulation within the brain. It is critical for maintaining synaptic plasticity, which supports learning, memory, and cognitive functions by facilitating structural and functional adaptations at synapses in response to new experiences. Additionally, its antioxidant and anti-inflammatory properties contribute to its neuroprotective effects, which may help counter neurodegenerative processes. Retinoic acid is vital for the development of the central nervous system, regulating the growth and differentiation of neurons and glial cells during embryonic development, and ensuring proper brain formation [77]. As an antioxidant, vitamin A helps neutralize free radicals, thereby protecting neurons from oxidative stress, a major contributor to neuronal damage and diseases like Alzheimer’s and Parkinson’s. Furthermore, it can modulate immune responses, reducing inflammation in the brain, which may slow the progression of Alzheimer’s disease. Impaired retinoic acid signaling is associated with increased accumulation of beta-amyloid plaques in Alzheimer’s disease. Some studies suggest that vitamin A supplementation may reduce beta-amyloid accumulation, and thus improve cognitive performance in AD patients [78]. In Parkinson’s disease, retinoic acid plays a role in regulating genes involved in dopamine production and neuron survival, potentially protecting against the degeneration of dopaminergic neurons. Vitamin A, due to its ability to reduce oxidative stress and increase dopamine production, has neuroprotective properties in Parkinson’s disease [79]. In multiple sclerosis, retinoic acid has been shown to reduce the activity of inflammatory T cells, which contribute to myelin damage. This action reduces neuroinflammation, promotes remyelination, and improves symptoms in patients with multiple sclerosis. Fragoso et al. suggest that vitamin A may reduce the rate of flare-ups, help regulate the immune system, and promote myelin repair. Thanks to this, it is possible to obtain better treatment results for patients with multiple sclerosis [80]. It is also important to remember that a significant body of evidence indicates that vitamin A concentrations over those required for normal cellular function can disrupt the redox environment, mitochondrial dysfunction, and induction of neuronal cell death [81]. A study by De Oliveira et al. found that supplementing vitamin A at doses ranging from 500 to 2500 IU/kg per day led to mitochondrial dysfunction and elevated levels of β-amyloid1-40 peptide and tumor necrosis factor-alpha (TNF-α) in the substantia nigra and striatum of adult rats. [82]. It has also been suggested that vitamin A supplementation may, at least in part, promote neuronal abnormalities and the increased rate of production of β-amyloid peptides in Alzheimer’s disease. Moreover, higher vitamin A consumption and elevated serum retinol levels have been associated with a greater risk of fractures and increased bone fragility in both humans and rodents [83]. However, data obtained from animal studies are insufficient to conclude that vitamin A supplementation is a risk factor for neurodegenerative diseases in humans [84].

#### 3.2.4. Vitamin K

Vitamin K is primarily recognized for its role in blood clotting, but it also has significant implications for brain health and has garnered interest for its potential neuroprotective effects. Diachenko et al. indicate that vitamin K may affect the development and progression of various neurodegenerative diseases through its involvement in antioxidant protection, sphingolipid metabolism, and inflammation regulation [85]. Recent studies have shown promising results regarding the effects of vitamin K2 in preventing apoptosis, oxidative stress, and microglial activation in neuronal cells through its role in electron transport, particularly in relation to Alzheimer’s disease [86]. Vitamin K is crucial for the metabolism of sphingolipids and plays essential roles in cell signaling and neuronal survival. Disruptions in sphingolipid metabolism have been associated with cognitive decline and neurodegenerative disorders, including Alzheimer’s disease [87]. Additionally, vitamin K possesses antioxidant properties that help shield neurons from oxidative stress, a significant factor in neuronal degeneration. Its anti-inflammatory effects may also help reduce neuroinflammation, potentially offering protection against the progression of Alzheimer’s and multiple sclerosis [88]. Some studies suggest that individuals with higher levels of vitamin K exhibit better cognitive function and memory performance. Postmortem studies of brain samples showed that the increased level of vitamin K2 correlated with the reduced risk of dementia, improved cognitive abilities, lower severity of AD, and lower level of phosphorylated Tau protein aggregation [89]. Research indicates that vitamin K may support the survival and growth of nerve cells, with some studies suggesting that it can reduce amyloid-beta deposition, thereby providing a protective effect against the progression of Alzheimer’s disease [90]. Furthermore, vitamin K may help protect dopaminergic neurons, similar to its protective effects observed in AD, by reducing oxidative damage and inflammation in the brain. Furthermore, vitamin K helps protect dopaminergic neurons, similar to its supportive effects observed in AD, by reducing oxidative damage and inflammation in the brain [89]. Beyond its protective impact on cognitive functions, vitamin K2 has demonstrated notable inhibitory effects on inflammation and the fibrillization of α-synuclein in Parkinson’s disease, as highlighted in recent findings [91,92]. Additionally, vitamin K has been suggested as a potential therapeutic option for multiple sclerosis patients to help reduce symptom severity or slow down the disease’s progression. By contributing to sphingolipid metabolism and providing antioxidant defense, vitamin K may protect nerve cells from degeneration in multiple sclerosis, potentially aiding in myelin repair and mitigating neuroinflammation [93,94,95].

## 4. The Authorized Health Claims Related to the Vitamins Relevant toNeurodegenerative Diseases

To effectively inform consumers about the discussed properties of vitamins, their positive health impacts, and their potential roles in preventing and supporting therapy for neurodegenerative diseases, health claims are displayed on food products and dietary supplements. To promote the use of vitamins in relation to neurodegenerative health, it is beneficial to review those health claims that are authorized for use in the European Union, which has a particularly comprehensive food law regulatory framework.

According to EU food law, a health claim is defined as “any claim that states, suggests, or implies that a relationship exists between a food category, a food, or one of its constituents and health” (Art. 2 (1)(5) of Regulation 1924/2006) [96]. Under the regulation, health claims are subject to preapproval, involving scientific assessment by the European Food Safety Authority (EFSA) [97]. EFSA verifies the supporting scientific studies, assessing their quality, safety, and effectiveness of the substance in question. “Health claims may be used in the labeling, presentation, and advertising of foods placed on the market in the Community only if they comply with the provisions of Regulation 1924/2006” (Art. 3 of Regulation 1924/2006) [96].

Most importantly, health claims must be substantiated by generally accepted scientific evidence (Art. 6 (1) of Regulation 1924/2006) [96]. The current list of authorized health claims is provided in the EU Register of Nutrition and Health Claims. This register enables food and dietary supplement producers to clearly communicate the health benefits associated with their products to consumers.

Table 1 provides a comprehensive list of all authorized claims for the vitamins discussed in the text. Table 2 presents an extract from this register, narrowed down to claims specific to the discussed vitamins, highlighting their recognized health effects, particularly those relevant to the prevention and support of neurodegenerative diseases. The tables were compiled based on data available in the EU Register of Health Claims [98]. The names of the vitamins analyzed in this study were entered into the search engine of the register. For each vitamin, authorized health claims accepted under EU regulations were recorded. The information included in the table comprises the health claim statement, indicating the impact of a given vitamin on bodily functions (e.g., “Riboflavin contributes to normal energy-yielding metabolism”), and the health relationship, providing the context of the vitamin’s action (e.g., “contribution to normal energy-yielding metabolism”).

Overall, the number of authorized health claims even remotely related to neurodegenerative diseases is relatively small. Furthermore, for some of the discussed vitamins, such as Vitamin B3, there are no authorized claims at all. This highlights the need to consider the reasons behind this state of affairs. It raises questions about whether the lack of authorized claims stems from insufficient research, the complexity of establishing direct links between these vitamins and neurodegenerative diseases, regulatory challenges in approving such claims, or perhaps a lack of market demand for such specific authorizations. As emphasized in “Can foods influence the onset and progress of neurodegenerative diseases?”, this presents a fascinating challenge for future research: developing foods and beverages rich in nutraceuticals with neuroprotective activity—targeting, for instance, energetic metabolism and oxidative stress—which could pave the way for new health claims and benefit the entire food chain, from raw material production to final product approval [99].

## 5. Conclusion

In conclusion, vitamins are vital for preventing and treating neurodegenerative diseases. Their varied roles—including antioxidant functions, anti-inflammatory properties, and the regulation of neurotransmitter production—underscore their potential as adjunct therapies in neurological conditions (Table 3). Key vitamins such as the B-complex group, vitamin C, vitamin D, vitamin E, vitamin A, and vitamin K are instrumental in preserving cognitive function, shielding the brain from oxidative stress, and fostering overall brain health. As ongoing research sheds light on how these vitamins provide neuroprotection, the significance of sufficient vitamin intake in lowering the risk of neurodegenerative disorders and aiding those already impacted becomes increasingly evident (Table 4). Further research is needed, as studies indicate that the metabolism of certain vitamins varies among individuals. A deeper understanding of the factors affecting their metabolism is essential for optimizing treatment outcomes. While personalizing vitamin supplementation can be costly, its potential benefits for individuals with neurodegenerative diseases and healthcare systems make it a promising area for future research. This growing body of evidence suggests that the currently limited number of authorized health claims linking vitamins to neurodegenerative disease prevention and therapy under Regulation 1924/2006 may expand in the future as scientific advancements progress. Emphasizing a balanced diet rich in essential vitamins, along with other lifestyle changes, can be an effective strategy for protecting brain health and improving the quality of life for both individuals with potential risk of occurrence and those who suffer from neurodegenerative diseases.

**Table 3:** Beneficial effects of vitamins on the brain.

Vitamin	Main Function in the Brain	Neuroprotective Effect	Nutritional Source	References
B_1_	Energy metabolism, neuronal function	Prevents cognitive decline, supports neuronal function	Whole grains, pork, sunflower seeds, pistachio nuts, soybeans, brown rice, legumes (lentils, beans)	[10,11,12,13,19,100,101]
B_2_	Energy metabolism, antioxidant defense, neurotransmitter regulation, and myelin health	Supports cognitive functions and visual perception, protects neurons from oxidative stress	Milk, liver, pork, beef, eggs, green leafy vegetables, almonds	[16,17,19,100,102]
B_3_	Energy metabolism, antioxidant defense, neurotransmitter regulation, anti-inflammatory	Supports mitochondrial function, reduces inflammation, protects neurons from oxidative damage, improves cognitive function	Poultry, fish (tuna, salmon), peanuts, macadamia nuts, pork, beef, mushrooms, whole grains	[19,21,22,23,24,25,100,102]
B_6_	Neurotransmitter synthesis (serotonin, dopamine, GABA), homocysteine metabolism	Reduces cognitive decline, supports mood and motor control, lowers homocysteine	Bananas, potatoes, poultry, fish, chickpeas	[10,19,26,28]
B_7_	Myelin synthesis, mitochondrial energy metabolism, gene expression	Supports remyelination, reduces inflammation, promotes neuron survival and energy metabolism	Egg yolks, nuts, seeds, salmon, dairy products, sweet potatoes	[19,31,32,33]
B_9_	DNA synthesis, repair, homocysteine regulation, neurotransmitter synthesis	Lowers homocysteine, prevents cognitive decline, supports neurodevelopment	Leafy greens (spinach, kale), citrus fruits, beans, peas	[19,35,36,37,38,39,41,42,45,100]
B_12_	DNA synthesis, myelin formation, homocysteine regulation, neurotransmitter regulation	Protects myelin, reduces oxidative stress, supports motor and cognitive function	Meat, fish, eggs, dairy products	[10,19,37,39,41,42,43,44,45,46,103]
D	Calcium homeostasis, neuroinflammation, neurotransmitter regulation	Reduces neuroinflammation, promotes neuronal survival, supports cognitive and motor function	Fatty fish (salmon, mackerel, sardines), egg yolks, butter	[58,59,60,61,62,63,64,102]
E	Antioxidant, neurotrophic factor support	Protects neurons from oxidative stress, promotes synaptic function, reduces inflammation	Nuts (almonds, sunflower seeds), spinach, avocado, vegetable oils (sunflower, safflower, wheat germ)	[67,69,71,72,73,78,104]
A	Synaptic plasticity, gene regulation, immune modulation	Reduces beta-amyloid plaques, supports dopamine production, reduces neuroinflammation	Plant sources (as beta-carotene): carrots, sweet potatoes, spinach, kale, mangoes, and apricots; liver, meat, fish, eggs, milk	[71,73,78,79,80]
C	Antioxidant, neurotransmitter synthesis, neuroprotection	Protects neurons from oxidative stress, supports cognitive function	Citrus fruits (oranges, lemons, grapefruits), strawberries, kiwi, bell peppers, broccoli, tomatoes	[49,50,51,52,54,56,57]
K	Sphingolipid metabolism, anti-inflammatory	Reduces inflammation, supports cognitive function	Green leafy vegetables (kale, spinach, broccoli), soybeans	[85,86,87,88,89,90,91,92,93,95,105]

**Table 4:** Vitamin supplementation in therapy of neurodegenerative diseases.

Vitamin	Key Findings	Reference
Vitamin A	Improves cognitive deficits in Alzheimer’s Disease patients	[106]
	Beneficial effect on reference memory	[107]
Vitamin D	Enhances cognitive performance and reduces Aβ-related biomarkers in Alzheimer’s Disease patients	[108]
	Prevents cognitive decline and improves spatial learning and memory in aging rats	[109]
Vitamin E	Lowers risk of Parkinson’s Disease	[73]
	Prevents the loss of dopaminergic neurons	[110]
	Beneficial effect on AD-related neuropathologic changes	[111]
Vitamin E + carotenoids + omega-3 fatty acids	Results in positive outcomes, reducing the severity of the disease in Alzheimer’s Disease patients	[71]
Vitamin B3 + B6 + B12 + Folate	Improves cognitive performance, decreases homocysteine levels, inhibits the expression of inflammatory factors	[37]
Vitamin B9 + B12	Improves cognitive function in patients with atrophy ratios higher than the median	[112]
Folic acid + vitamin B6 + vitamin B12	Slows the atrophy of specific brain regions that are a key component of the Alzheimer’s Disease process and that are associated with cognitive decline	[113]
Vitamin C	Decreases the accumulation of amyloid plaques; improves the degenerative alterations in the brain of Alzheimer’s Disease mice, leading to improvement in blood–brain barrier disruption and changes in mitochondrial function	[56]
Vitamin C + E	Reduces the vulnerability of CSF and plasma lipoproteins to oxidation	[114]
	Significant effect on the course of Alzheimer’s Disease over 1 year of supplementation	[115]
K	Reduces neuronal deterioration and cognitive loss	[116]

## Figures and Tables

**Table 1:** List of authorized health claims related to the discussed vitamins relevant to neurodegenerative diseases.

Vitamin	Claim	Health Relationship
Vitamin B_2_	Contributes to normal functioning of the nervous system	Maintenance of the normal function of the nervous system
	Contributes to the protection of cells from oxidative stress	Protection of DNA, proteins, and lipids from oxidative damage
	Contributes to the reduction of tiredness and fatigue	Reduction of tiredness and fatigue
Vitamin B_12_	Contributes to normal functioning of the nervous system	Contribution to neurological and psychological function
	Contributes to normal homocysteine metabolism	Contribution to normal homocysteine metabolism
	Contributes to normal psychological function	Contribution to neurological and psychological function
Vitamin B_6_	Contributes to normal homocysteine metabolism	Contribution to normal homocysteine metabolism
	Contributes to normal psychological function	Contribution to normal psychological functions
	Contributes to normal functioning of the nervous system	Function of the nervous system
Vitamin D	Contributes to the normal function of the immune system	Normal function of the immune system and inflammation response
Vitamin E	Contributes to the protection of cells from oxidative stress	Protection of DNA, proteins, and lipids from oxidative damage
Vitamin C	Contributes to the protection of cells from oxidative stress	Protection of DNA, proteins, and lipids from oxidative damage
	Contributes to normal functioning of the nervous system	Function of the nervous system
	Contributes to normal psychological function	Contribution to normal psychological functions

**Table 2:** List of all authorized health claims related to the discussed vitamins.

Vitamin	Claim	Health Relationship
Vitamin B_2_	Contributes to normal energy-yielding metabolism	Contribution to normal energy-yielding metabolism
	Contributes to normal functioning of the nervous system	Maintenance of the normal function of the nervous system
	Contributes to the maintenance of normal mucous membranes	Maintenance of normal skin and mucous membranes
	Contributes to the maintenance of normal red blood cells	Maintenance of normal red blood cells
	Contributes to the maintenance of normal skin	Maintenance of normal skin and mucous membranes
	Contributes to the maintenance of normal vision	Maintenance of normal vision
	Contributes to the normal metabolism of iron	Contribution to normal metabolism of iron
	Contributes to the protection of cells from oxidative stress	Protection of DNA, proteins, and lipids from oxidative damage
	Contributes to the reduction of tiredness and fatigue	Reduction of tiredness and fatigue
Vitamin B_12_	Contributes to normal energy-yielding metabolism	Energy-yielding metabolism
	Contributes to normal functioning of the nervous system	Contribution to neurological and psychological function
	Contributes to normal homocysteine metabolism	Contribution to normal homocysteine metabolism
	Contributes to normal psychological function	Contribution to neurological and psychological function
	Contributes to normal red blood cell formation	Red blood cell formation
	Contributes to the normal function of the immune system	Function of the immune system
	Contributes to the reduction of tiredness and fatigue	Reduction of tiredness and fatigue
	Has a role in the process of cell division	Cell division
Vitamin B_6_	Contributes to normal homocysteine metabolism	Contribution to normal homocysteine metabolism
	Contributes to normal protein and glycogen metabolism	Protein and glycogen metabolism
	Contributes to normal psychological function	Contribution to normal psychological functions
	Contributes to normal red blood cell formation	Red blood cell formation
	Contributes to the normal function of the immune system	Function of the immune system
	Contributes to the reduction of tiredness and fatigue	Reduction of tiredness and fatigue
	Contributes to the regulation of hormonal activity	Regulation of hormonal activity
	Contributes to normal cysteine synthesis	Contribution to normal cysteine synthesis
	Contributes to normal energy-yielding metabolism	Contribution to normal energy-yielding metabolism
	Contributes to normal functioning of the nervous system	Function of the nervous system
Calcium and Vitamin D	Needed for normal growth and development of bone in children	-/-
	Help to reduce loss of bone mineral in post-menopausal women	-/-
Vitamin D	Needed for normal growth and development of bone in children	-/-
	Helps reduce the risk of falling associated with postural instability and muscle weakness	-/-
	Contributes to normal absorption/utilization of calcium and phosphorus	Absorption and utilization of calcium and phosphorus, maintenance of blood calcium levels
	Contributes to normal blood calcium levels	Absorption and utilization of calcium and phosphorus, maintenance of blood calcium levels
	Contributes to the maintenance of normal bones	Maintenance of bones and teeth
	Contributes to the maintenance of normal muscle function	Normal muscle function
	Contributes to the maintenance of normal teeth	Maintenance of bones and teeth
	Contributes to the normal function of the immune system	Normal function of immune system and inflammation response
	Has a role in the process of cell division	Cell division
	Contributes to the normal function of the immune system in children	-/-
Vitamin E	Contributes to the protection of cells from oxidative stress	Protection of DNA, proteins, and lipids from oxidative damage
Vitamin A	Contributes to normal iron metabolism	-/-
	Contributes to the maintenance of normal mucous membranes	Maintenance of normal skin and mucous membranes
	Contributes to the maintenance of normal skin	Maintenance of normal skin and mucous membranes
	Contributes to the maintenance of normal vision	Maintenance of normal vision
	Contributes to the normal function of the immune system	Maintenance of the normal function of the immune system
	Has a role in the process of cell specialization	Cell differentiation
Vitamin K	Contributes to normal blood clotting	Blood coagulation
	Contributes to the maintenance of normal bones	Maintenance of bones
Vitamin C	Contributes to maintain the normal function of the immune system during and after intense physical exercise	Function of the immune system during and after extreme physical exercise
	Contributes to normal collagen formation for the normal function of blood vessels	Collagen formation
	Contributes to normal collagen formation for the normal function of bones	Collagen formation
	Contributes to normal collagen formation for the normal function of cartilage	Collagen formation
	Contributes to normal collagen formation for the normal function of gums	Collagen formation
	Contributes to normal collagen formation for the normal function of skin	Collagen formation
	Contributes to normal collagen formation for the normal function of teeth	Collagen formation
	Contributes to normal energy-yielding metabolism	Contribution to normal energy-yielding metabolism
	Contributes to normal functioning of the nervous system	Function of the nervous system
	Contributes to normal psychological function	Contribution to normal psychological functions
	Contributes to the normal function of the immune system	Maintenance of the normal function of the immune system
	Contributes to the protection of cells from oxidative stress	Protection of DNA, proteins, and lipids from oxidative damage
	Contributes to the reduction of tiredness and fatigue	Reduction of tiredness and fatigue
	Contributes to the regeneration of the reduced form of vitamin E	Regeneration of the reduced form of vitamin E
	Increases iron absorption	Non-haem iron absorption

## Data Availability

No new data were created or analyzed in this study. Data sharing is not applicable to this article.
